# Isolation, Identification and Whole-Genome Sequencing of a *Nocardia seriolae* Strain from Farmed Chinese Rice-Field Eels (*Monopterus albus*)

**DOI:** 10.3390/ani16081160

**Published:** 2026-04-10

**Authors:** Wenzhi Liu, Hongyang Song, Anda Cheng, Chu Ma, Xin Ren, Yiqun Li, Hao Wang, Nan Jiang, Yong Zhou, Rui Ruan, Yuding Fan

**Affiliations:** 1 Yangtze River Fisheries Research Institute, Chinese Academy of Fishery Sciences, Wuhan 430223, China; liuwenzhialisa@yfi.ac.cn (W.L.); 15302841497@163.com (H.S.); 19848177792@163.com (C.M.); renxin@yfi.ac.cn (X.R.); liyq@yfi.ac.cn (Y.L.); jn851027@yfi.ac.cn (N.J.); zhouy@yfi.ac.cn (Y.Z.); 2 College of Fisheries and Life Science, Shanghai Ocean University, Shanghai 201306, China; h-wang@shou.edu.cn; 3 Beijing Centre Technology Co., Ltd., Beijing 102600, China; cheng.anda@163.com

**Keywords:** Chinese rice-field eels (*Monopterus albus*), *Nocardia seriolae* strain JXMa251025, whole-genome sequencing, pathogenicity, antibiotic susceptibility

## Abstract

In October 2025, an outbreak occurred among farmed Chinese rice-field eels (*Monopterus albus*) in Jiangxi, China. A *Nocardia seriolae* strain designated JXMa251025, which has not been previously documented in Chinese rice-field eels, was isolated from moribund fish exhibiting multiple white nodules of various sizes in visceral tissues. Histopathological examination revealed multi-organ damage, including necrosis of liver cells, granulomatous inflammation with hemorrhage in visceral organs, and necrosis of renal glomeruli and tubules accompanied by vascular congestion. Artificial infection trials confirmed that strain JXMa251025 reproduced clinical signs consistent with those observed in the natural outbreak. Infection experiments resulted in 100% mortality in high-concentration challenge groups, with a median lethal dose (LD_50_) of 9.76 × 10^5^ CFU/mL, indicating high virulence. Whole-genome sequencing revealed a circular chromosome of 8,295,032 bp with a GC content of 68.10%. The genome contains 66 tRNA genes and four copies each of the 23S, 16S, and 5S rRNA genes. Phylogenomic analysis placed strain JXMa251025 within a clade of *Nocardia seriolae* strains with approximately 99% bootstrap support, confirming its identification as *Nocardia seriolae*. Further genomic screening identified 253 potential virulence genes associated with nutrient metabolism, regulatory systems, immune modulation, effector delivery, and exotoxin production. Antibiotic susceptibility testing showed that strain JXMa251025 was sensitive to seven antibiotics: ciprofloxacin, neomycin, enrofloxacin, florfenicol, gentamicin, amikacin, and doxycycline. This study represents the first report of *Nocardia seriolae* infecting Chinesse rice-field eels, providing useful descriptive information for disease diagnosis and reference.

## Simple Summary

The Chinese rice-field eel (*Monopterus albus*) is an important freshwater fish widely cultured in China. In recent years, disease outbreaks have caused heavy economic losses to its aquaculture. In October 2025, an outbreak of severe disease with high mortality occurred among farmed individuals in Jiangxi Province. Infected fish showed obvious white nodules in visceral organs and severe tissue lesions. This study first isolated *Nocardia seriolae* from diseased individuals and confirmed it as the causative pathogen. Artificial infection experiments verified its high pathogenicity to the Chinese rice-field eel. We also analyzed its genomic characteristics and antibiotic susceptibility profile. These results provide useful descriptive information and reference for the diagnosis and identification of this disease in the Chinese rice-field eels.

## 1. Introduction

The Chinese rice-field eel (*Monopterus albus*), a member of the *Synbranchidae* family, is a commercially significant freshwater fish species widely distributed across East and Southeast Asia [1]. Valued for its delicate flavor, nutritional richness, and purported medicinal benefits, it has become a highly sought-after commodity, driving the rapid expansion of its aquaculture industry in China [2]. According to recent fishery statistics, annual farmed production has exceeded 300,000 tons, underscoring its substantial role in the domestic aquaculture sector [3]. However, this growth has been paralleled by an increase in disease outbreaks, which now pose a constraint on the industry’s sustainability. High mortality rates persist across both larval and adult stages, attributed to complex factors including suboptimal water quality, high stocking densities, and infectious diseases [4,5,6,7]. In the present, well-documented pathogens have been reported, such as *Aeromonas veronii* [8], *Aeromonas hydrophila* [7,9], rhabdovirus [6,10,11], *Edwardsiella tarda* [12], and *Entamoeba chiangraiensisn.* sp. [5]; however, several emerging pathogens have recently been identified as causes of significant mortality in farmed channel catfish in China.

In October 2025, a highly contagious and lethal disease outbreak emerged among farmed Chinese rice-field eels in Jiangxi Province, China. The outbreaks occurred at water temperatures between 22 °C and 25 °C. Affected Chinese rice-field eels exhibited clinical signs including anorexia, loss of equilibrium, and erratic swimming behavior. External examination revealed a swollen anus and ulceration lesions on the body surface. Necropsy findings showed multifocal, variably sized white nodules distributed throughout the liver, spleen, kidneys, and intestinal tract. Attempts to detect a known viral pathogen—including rhabdovirus—proved negative. Given the pathological presentation of visceral granulomas, which resembles that described in previous reports of aquatic nocardiosis [13,14], bacterial isolation and identification were initiated from affected tissues to establish a definitive etiology. The findings suggest the involvement of a potentially novel bacterial agent not previously reported in Chinese rice-field eels.

To address this issue, the present study was conducted to comprehensively characterize a *Nocardia* pathogen isolated from diseased Chinese rice-field eels in China. We performed bacterial isolation and identification, experimental infection trials, antibiotic susceptibility testing, whole-genome sequence analysis, and screening of virulence-associated genes. The isolated strain, designated JXMa251025, was confirmed as the etiological agent responsible for the outbreak affecting Chinese rice-field eels in Jiangxi Province, China. Complete genome sequencing revealed that the pathogen shares approximately 99% nucleotide sequence identity with *Nocardia seriolae*. Phylogenetic analysis based on whole-genome coding sequences indicated that JXMa251025 and *Nocardia seriolae* form a distinct monophyletic clade within the family *Nocardia* spp., suggesting a potential risk of cross-species transmission of *Nocardia seriolae* among farmed aquatic species. These findings provide important insights for disease surveillance and health management in the rapidly expanding Chinese rice-field eel aquaculture industry.

## 2. Materials and Methods

### 2.1. Experimental Chinese Rice-Field Eels

In October 2025, moribund Chinese rice-field eels displaying multifocal, variably sized white nodules on visceral surfaces were collected from a commercial aquaculture facility in Jiangxi Province, China. The affected individuals (mean body length 35.0 ± 0.3 cm) were transported to the laboratory of the Yangtze River Fisheries Research Institute, Chinese Academy of Fishery Sciences for comprehensive pathogen identification. For subsequent infection experiments, approximately 200 clinically healthy Chinese rice-field eels (30–35 cm in length) were obtained from the farm in Xiantao, Hubei province. Prior to the experiment, pathological changes were checked; Chinese rice-field eels were anesthetized and dissected, and their gill and liver tissues were tested for Chinese rice-field eel rhabdovirus (CrERV) and pathogenic bacteria (*Nocardia* spp., *Aeromonas* spp., *Edwardsiella tarda*, *Vibrio* spp.), with all results negative. Following confirmation of pathogen-free status, the Chinese rice-field eels were randomly divided into four experimental groups and a control group, with 30 fish in each. All specimens were maintained in flow-through tanks (2 m × 1.5 m × 1.0 m) with continuous aeration, under a controlled water temperature of 25.0 ± 0.5 °C. Dechlorinated municipal tap water was used, and key water-quality parameters were monitored daily. During a 14-day acclimatization period, the fish were fed a formulated diet consisting of *Limnodrilus* and minced fish meat.

### 2.2. Histopathological Sample Preparation

Tissue samples (spleen, liver, kidney, and intestine) were collected from both diseased and healthy Chinese rice-field eels and fixed in 4% paraformaldehyde at 4 °C for 24 h. After fixation, the samples were rinsed in Dulbecco’s phosphate-buffered saline (DPBS; Sigma, Kawasaki, Japan), trimmed, and transferred to embedding cassettes. Tissue dehydration was carried out through a graded ethanol series (45%, 60%, 75%, 85%, 95%, and 100%), followed by clearing in xylene and embedding in paraffin. Sections of 4–5 μm thickness were prepared using a rotary microtome and stained with hematoxylin and eosin (H&E) [15]. Histopathological evaluation was performed under a light microscope (DM2500; Leica Microsystems, Wetzlar Germany).

### 2.3. Bacterial Isolation

Diseased Chinese rice-field eels were euthanized with 0.1% tricaine methanesulfonate (MS-222; Sigma, Kawasaki, Japan) and surface-sterilized with 75% ethanol prior to dissection. Liver and kidney tissues were aseptically collected and individually streaked onto tryptic soytone agar (TSA) (HopeBio, Qingdao, China) plates. The plates were incubated at 28 °C for 7 days. After incubation, dominant and morphologically uniform bacterial colonies were selected and repeatedly streaked on fresh TSA plates for purification. Single colonies were finally isolated and stored in glycerol at −80 °C for further characterization.

### 2.4. Morphological Observation of Bacterial Isolates

For morphological characterization of the bacterial isolates, pure colonies were suspended in DPBS buffer, smeared onto glass slides, dried, and Gram-stained using a commercial kit (Gram Stain Kit; Jiancheng, Nanjing, China) in accordance with the manufacturer’s instructions. An optical microscope (Olympus, Tokyo, Japan) was used to observe the staining results and bacterial morphological characteristics. To further analyze the microscopic morphology of the bacteria, a 2.5% glutaraldehyde solution was used to fix the bacterial samples for 24 h at 4 °C. Subsequently, the samples were dehydrated and dried, and then observed under a scanning electron microscope (Hitachi, Tokyo, Japan) [16].

### 2.5. Identification of the Bacteria Isolates

The identification of bacteria isolates was performed through 16S rRNA gene sequencing. Genomic DNA of bacteria isolates was extracted with a Bacterial Genomic DNA Kit (Tiangen, Beijing, China) following the manufacturer′s instructions. The purified DNA served as the template for PCR amplification. Primers were designed as previously described [17] (Table 1). The PCR protocol consisted of an initial denaturation at 95 °C for 5 min, followed by 35 cycles of denaturation at 94 °C for 30 s, annealing at 55 °C for 45 s, and extension at 72 °C for 60 s, with a final extension at 72 °C for 7 min. The amplified products were separated by electrophoresis on a 1.5% agarose gel and visualized after ethidium bromide staining. The phylogenetic tree was constructed based on the obtained 16S rRNA sequence using MEGA V7.0.

To further identify the bacterial isolates, their biochemical characteristics were analyzed using microbial miniaturized biochemical test tubes (HopeBio, Qingdao, China) and a biochemical coding identification kit for non-fermenting bacteria (HuanKai Microbial, Guangzhou, China). The assay panel included tests for esculin, sorbitol, mannitol, urea, arabinose, fructose, galactose, lactose, sucrose, glucose, maltose, citrate, lysine, ornithine, gelatine, catalase and nitrate reduction.

### 2.6. Whole Bacterial Genome Sequencing, Assembly and Annotation

Genomic DNA of *Nocardia seriolae* strain JXMa251025 was isolated with the Qiagen DNeasy Blood & Tissue kit (Qiagen, Hilden, Germany) following the supplier’s protocol. Whole-genome sequencing was performed on a PacBio RS II platform (Pacific Biosciences, Menlo Park, CA, USA) using single-molecule real-time (SMRT) technology. For this purpose, a 20 kb SMRTbell library was constructed and sequenced with P6 polymerase/C4 chemistry on a dedicated SMRT cell. De novo assembly and circularization of the resulting reads were carried out with HGAP2 within the SMRTpipe pipeline and the Proksee (https://proksee.ca/), respectively. Predicted protein-coding genes were identified using Glimmer 3.02, followed by functional annotation through homology searches against the NCBI non-redundant protein (NR), Gene Ontology (GO), Clusters of Orthologous Groups (COG), and Kyoto Encyclopedia of Genes and Genomes (KEGG) databases.

### 2.7. Phylogenetic Analysis

The bacterial sequences acquired in this investigation were aligned with reference sequences of other 24 of *Nocardia* pathogens obtained from GenBank (Table 2). ANIb was analyzed online using JspeciesWS (https://jspecies.ribohost.com/jspeciesws/#analyse, accessed on 3 January 2026) for species identification and phylogenetic relationship analysis of strain JXMa251025. The GBDP phylogenetic tree of whole-genome coding sequences of JXMa251025 was constructed via the FastME 2.1.6.1 tool in TYGS (https://tygs.dsmz.de/, accessed on 26 December 2025) with whole-genome sequences of other *Nocardia* spp.

### 2.8. Infection Experiment

To assess the pathogenicity of the bacterial isolate, an experimental infection was carried out using clinically healthy Chinese rice-field eels sourced from a farm in Xiantao City, Hubei Province, China. Chinese rice-field eels were randomly divided into five groups (30 fish per group) for pathogen challenge. Each experimental group was intraperitoneally injected with 0.1 mL of bacterial suspension washed three times with PBS, at concentrations of 2.6 × 10^5^, 2.6 × 10^6^, 2.6 × 10^7^, and 2.6 × 10^8^ CFU/mL, respectively. Control fish received an equal volume (0.1 mL) of PBS via the same route. Throughout the experiment, all fish were kept in aerated water maintained at 25 °C. Clinical symptoms and mortality were monitored daily. From each group—including the controls—three moribund or deceased fish were randomly selected for PCR analysis to verify bacterial presence. The median lethal dose (LD_50_) of the bacterial isolate for Chinese rice-field eels was calculated using the Reed–Muench method.

### 2.9. Susceptibility to Antibiotics Assay

The antibiotic susceptibility of bacterial isolate was assessed in triplicate by the standard disk diffusion method, testing the following agents: ciprofloxacin, cefixime, neomycin, enrofloxacin, florfenicol, gentamicin, amikacin, sulfamethazine, sulfafurazole and doxycycline. Briefly, the bacterial isolate was cultured in TSA medium for 72 h. The bacterial suspension was then adjusted to 1 × 10^8^ CFU/mL with sterile PBS and evenly spread onto TSA plates. Before the inoculum dried, antibiotic-impregnated disks (Hangwei, Beijing, China) were aseptically placed on the agar surface. Plates were incubated at 28 °C for 96 h in a constant-temperature incubator (CIMO, Shijiazhuang, China). After incubation, the diameter of the inhibition zone around each disk was measured, and susceptibility was interpreted according to the manufacturer’s criteria.

## 3. Results

### 3.1. Clinical Symptoms

**Figure 1:**
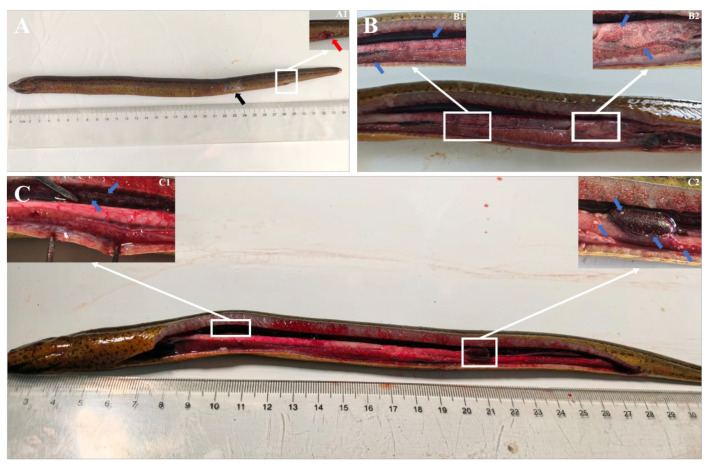
Clinical and pathological manifestations in infected Chinese rice-field eels. (**A**) External signs include skin ulceration (black arrow) and (**A1**) Close-up view of anal hyperemia and swelling (red arrow). (**B**) Dissected abdomen revealing multiple variably sized white nodules distributed in (**B1**) the liver and (**B2**) the intestine. (**C**) White nodules present in (**C1**) the kidney and (**C2**) the spleen. Blue arrow: white nodules.

Diseased fish exhibited characteristic clinical signs such as anorexia, erratic swimming patterns, skin ulcerations, as well as anal hyperemia and swelling (Figure 1A,A1). Upon necropsy, visceral congestion and multiple white nodules of diverse sizes were evident. These lesions were widespread in the liver (Figure 1B,B1) and intestine (Figure 1B,B2); the kidney (Figure 1C,C1) and spleen (Figure 1C,C2) were also obvious. Microscopic evaluation of skin, intestine and external mucus samples showed no evidence of parasitic infestation. In addition, our results indicate an absence of detectable infection by any of the targeted known viral pathogens associated with the observed clinical manifestations.

### 3.2. Histopathological Analysis

**Figure 2:**
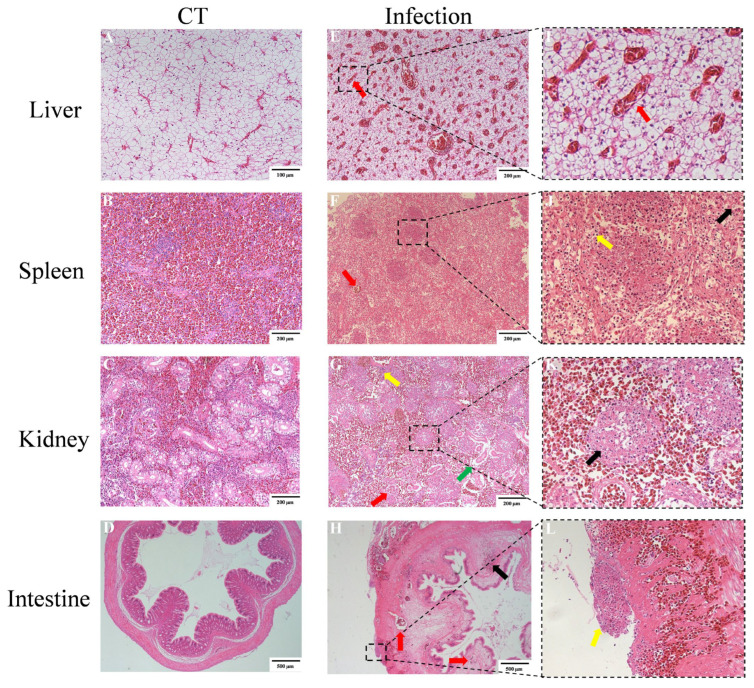
Histopathology of tissues from naturally and artificially infected Chinese rice-field eels. Tissues from healthy control Chinese rice-field eels: liver (**A**), spleen (**B**), kidney (**C**), and intestine (**D**). (**E**–**H**) Tissues from naturally infected individuals: (**E**) liver, showing enlarged hepatic sinusoids and blood vessels (red arrow); (**F**) spleen, exhibiting extensive necrosis and inflammatory cell infiltration throughout the organ (yellow arrow), the presence of melanomacrophage centers (red arrow), and necrotic splenocytes with pale-stained, marginated nuclei (black arrow); (**G**) kidney, demonstrating widespread erythrocyte infiltration and severe necrosis (red arrow), inflammatory cell infiltration in the hematopoietic tissue (yellow arrow), necrotic hematopoietic cells with marginated nuclei (black arrow), severe edema of renal tubules (red arrow), and necrotic glomeruli with erythrocyte infiltration (green arrow); (**H**) intestine, revealing enlarged vessels (red arrow), erythrocyte and inflammatory cell infiltration in both the smooth muscle and submucosal layers (black arrow), and granulomatous lesions on the serosa with mild inflammatory infiltration (yellow arrow); (**I**–**L**) enlarged image of typical clinical signs.

Naturally diseased and artificially infected Chinese rice-field eels exhibited distinct pathological changes in the liver, spleen, kidney, and intestine. In contrast, no significant histological alterations were observed in the corresponding organs of healthy control fish (Figure 2A–D). In the liver of the infected fish, histopathological analysis showed enlarged hepatic sinusoids and blood vessels (Figure 2E). The infected spleen showed extensive necrosis and inflammatory cell infiltration of the entire organ, as well as the presence of the melanomacrophage centers. The necrotic splenocytes presented pale stained and marginated nuclei (Figure 2F). The infiltration of erythrocyte and severe necrosis were distributed throughout the infected kidney. The inflammatory cell infiltration was detected in the hematopoietic tissue, and the necrotic hematopietic tissue cells showed marginated nuclei. Serious edema renal tubulars were observed, and the glomerulus showed necrosis and erythrocyte infiltration (Figure 2G). Histopathological examination of the infected intestinal tissue revealed enlarged vessels and erythrocyte and inflammatory cell infiltration in both the smooth muscle layer and submucosal layer. Furthermore, granulomatous lesions were observed on the membranse serosa layer (Figure 2H).

### 3.3. Morphological Characterization of JXMa251025

**Figure 3:**
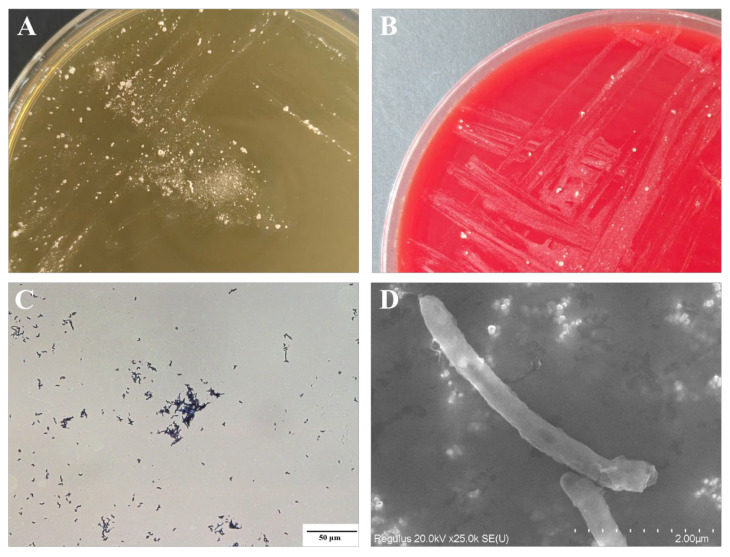
Morphological characteristics of *Nocardia seriolae* (strain JXMa251025). (**A**) Colonies grown on TSA at 28 °C for 7 days, showing white, sandy-granular appearance with irregular margins. (**B**) Colony morphology after 3 days of incubation on sheep blood agar at 28 °C. (**C**) Gram staining of isolate JXMa251025 (scale bar: 50 µm). (**D**) Scanning electron micrograph of isolate JXMa251025 (scale bar: 2.0 µm).

The isolated primary pathogen exhibited slow growth on TSA medium at 28 °C, requiring approximately 5–7 days for colony formation. Macroscopic observation revealed white, sandy-granular colonies with irregular margins (Figure 3A). In contrast, the bacterium grew rapidly on sheep blood agar, reaching a comparable colony size after only 3 days of incubation at 28 °C (Figure 3B). During subculture, colonies were notably firm and difficult to detach and manipulate once removed from the solid medium. Gram staining indicated that the strain was Gram-positive (appearing blue-purple) and consisted of non-sporulating rods or branching filaments under light microscopy (Figure 3C). To further characterize its ultrastructure, the purified strain JXMa251025 was examined using scanning electron microscopy. The results showed that JXMa251025 displayed a branched, rod-shaped morphology. Bacterial cells adhered tightly to one another, appearing as straight or slightly curved rods without visible flagella. The dimensions of JXMa251025 ranged from 5 to 8 μm in length and 0.50 to 0.58 μm in width (Figure 3D).

### 3.4. Genomic Features and Functional Annotation of Strain JXMa251025

**Figure 4:**
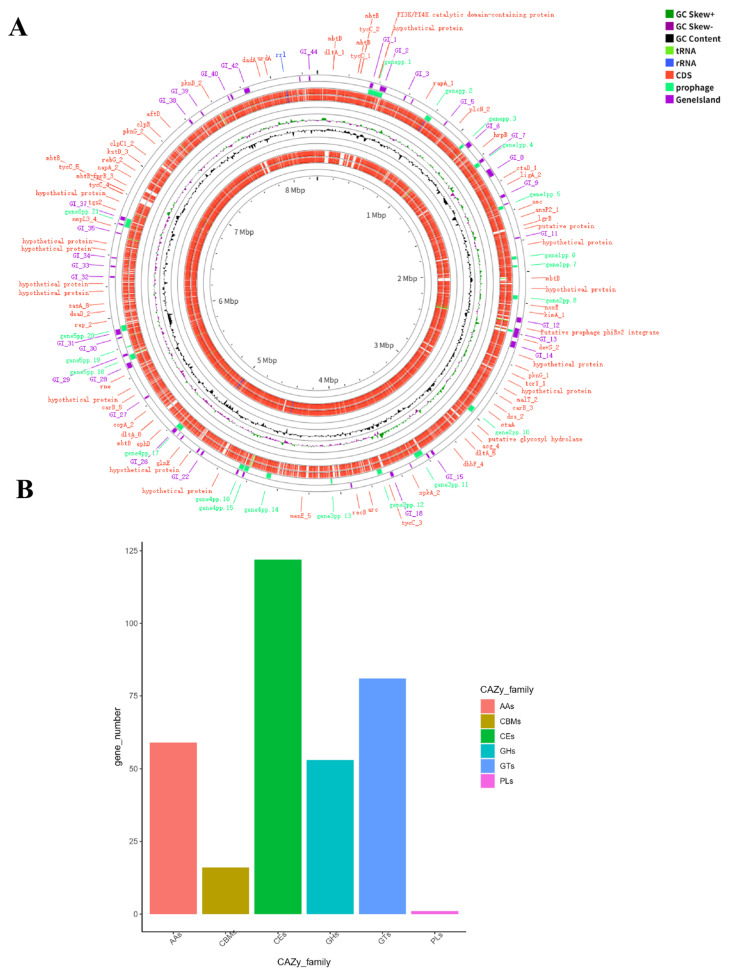
Circular genome map of *Nocardia seriolae* JXMa251025 and distribution of CAZy enzyme families. (**A**) The complete genome is represented with concentric circles displaying: outermost, 20 kb scale marks; second circle, protein-coding genes on the forward strand. (**B**) Bar plot showing the number of distinct CAZy families per functional category. The Auxiliary Activities (AAs) category contains the highest family diversity (59), exceeding all other categories, while Polysaccharide Lyases (PLs) have only one family. CAZy categories are: AAs, Carbohydrate-Binding Modules (CBMs, 16), Carbohydrate Esterases (CEs, 122), Glycoside Hydrolases (GHs, 53), Glycosyl Transferases (GTs, 81), and PLs (1).

After assembly, the genome of this *Nocardia* JXMa251025 strain has a total length of 8,295,032 bp with a GC content of 68.10%. It contains 66 transfer RNA genes, as well as four copies each of the 23S, 16S, and 5S ribosomal RNA (rRNA) genes. Bioinformatic predictions identified 44 gene islands and 21 prophages in the genome (Figure 4A). The results of the CAZy database annotation analysis of carbohydrate-active enzyme genes revealed that the JXMa251025 genome contains 59 auxiliary redox enzymes (AAs), 16 non-catalytic carbohydrate-binding modules (CBMs), 122 carbohydrate esterases (CEs), 53 glycoside hydrolases (GHs), 81 glycosyltransferases (GTs), and 1 polysaccharide lyase (PL) (Figure 4B).

**Figure 5:**
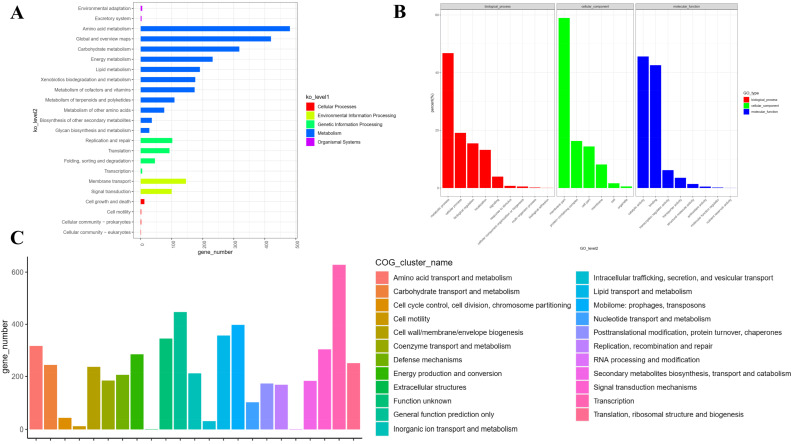
Functional annotation of predicted genes in *Nocardia seriolae* strain JXMa251025 genome across KEGG, GO, and COG databases. (**A**) KEGG pathway classification. Bars represent the number of genes assigned to each pathway category (values shown above bars). (**B**) Gene Ontology (GO) enrichment analysis. Significantly enriched terms (metabolic process, membrane part and catalytic activity) are highlighted, reflecting core biological process, cellular component and molecular function. (**C**) Clusters of Orthologous Groups (COG) classification. Predicted protein-coding genes are categorized into functional classes based on orthology.

KEGG pathway annotation revealed a versatile and comprehensive metabolic repertoire (Figure 5A). The genome was significantly enriched in genes associated with environmental adaptation and membrane transport, underscoring its capacity to thrive under diverse selective pressures. Core metabolic pathways for carbohydrate, amino acid, lipid, and energy metabolism were extensively represented, indicating a broad nutritional utilization spectrum. Furthermore, complete modules for genetic information processing (replication, transcription, translation) and cellular processes were identified, supporting robust growth and regulatory functions.

Gene Ontology (GO) enrichment analysis of the gene set revealed a strong functional bias toward core metabolic and catalytic activities. The majority of genes were associated with metabolic processes (1477 genes) within the Biological Process domain. In Cellular Component, annotations overwhelmingly mapped to protein-containing complexes (464 genes), indicating prevalent involvement in multiprotein assemblies. For Molecular Function, catalytic activity (1291 genes) represented the dominant functional category. Additional notable functions—each comprising 1341 genes—included biological regulation, localization, transport activity, and antibiotic activity, suggesting specialized adaptive capabilities alongside the core metabolic focus (Figure 5B).

Functional annotation based on Clusters of Orthologous Groups (COG) revealed a distinct genomic profile characterized by strong investment in core metabolic processes. The categories “Amino acid transport and metabolism” (317 genes) and “Carbohydrate transport and metabolism” (244 genes) were the most abundant, indicating a high metabolic capacity (Figure 5C). Association analysis between COG categories further showed coordinated functional architecture. Key metabolic categories (amino acid and carbohydrate metabolism) were closely linked, suggesting integrated nutrient utilization. Additionally, cellular processes (“Cell wall/membrane biogenesis,” “Cell motility,” “Cell cycle control”) displayed notable interconnectivity, supporting coordinated growth and environmental interaction. The “Defense mechanisms” category also showed specific associations with transport systems, hinting at adaptive stress responses.

### 3.5. Virulence Factor Analysis

**Figure 6:**
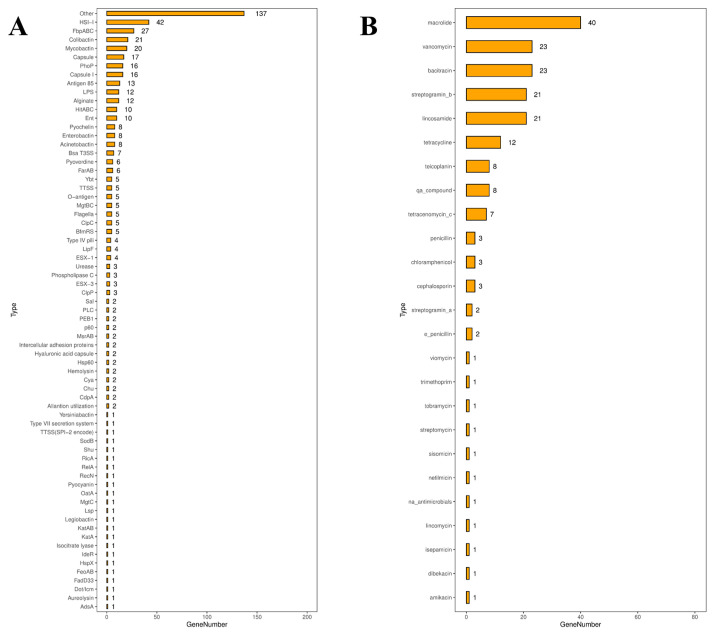
Distribution of virulence-associated gene counts and antibiotic resistance gene in *Nocardia seriolae* strain JXMa251025. (**A**) Virulence factor analysis in *Nocardia seriolae* strain JXMa251025. (**B**) Antibiotic resistance gene analysis in *Nocardia seriolae* strain JXMa251025.

Based on the genetic analysis, the bacterial isolate displays a comprehensive and significant virulence-associated gene profile indicative of a potentially high-pathogenicity strain (Figure 6A). The most dominant features are genes involved in iron acquisition, particularly the highly abundant HSI-I (42) and FbpABC (27) systems, supported by multiple siderophore clusters such as Mycobactin (20), Colibactin (21), Pyochelin (8), Enterobactin (8), and Acinetobactin (8), underscoring a robust capacity for iron scavenging essential for in vivo survival and growth. Concurrently, a strong repertoire of genes related to immune evasion and surface integrity is present, including Capsule (17), Capsule I (16), LPS (12), Alginate (12), and Antigen 85 (13), which likely contribute to resistance against host defenses. Additionally, multiple secretion system genes (Bsa T3SS (7), TTSS (5), ESX-1 (4)) suggest active mechanisms for effector delivery, while other key virulence determinants such as PhoP (16), Flagella (5), Type IV pilus (4), Urease (3), Phospholipase C (3), and Hemolysin (2) further reflect adaptability in adhesion, motility, regulation, and tissue damage.

### 3.6. Antibiotic Resistance Gene Analysis

Genomic sequencing of strain JXMa251025 identified a diverse and abundant reservoir of antibiotic resistance determinants. The isolate′s resistome encompassed genes conferring potential resistance to 14 distinct antimicrobial classes (Figure 6B). Macrolide resistance genes were the most numerous (*n* = 40), followed by a notable repertoire of glycopeptide resistance determinants, including those for vancomycin (*n* = 23) and teicoplanin (*n* = 8). Substantial genetic capacities for resistance were also observed against bacitracin (*n* = 23), streptogramins (type B, *n* = 21; type A, *n* = 2), and lincosamides (*n* = 21). Furthermore, the genome carried multiple genes associated with resistance to tetracyclines (*n* = 12), beta-lactams (penicillins, *n* = 3; cephalosporins, *n* = 3), and chloramphenicol (*n* = 3). A variety of additional resistance genes, present in lower copy numbers, were detected that may confer reduced susceptibility to aminoglycosides (tobramycin, amikacin, streptomycin), trimethoprim, and other antimicrobial classes (Figure 6B).

### 3.7. Antibiotic Susceptibility Analysis of JXMa251025

Antibiotic susceptibility was assessed using the disc diffusion method. The inhibition zone data, presented as mean ± standard deviation, are summarized in Table 3. The results indicate that the isolated strain JXMa251025 was sensitive to seven of the ten antibiotics tested, including ciprofloxacin, neomycin, enrofloxacin, florfenicol, gentamicin, amikacin, and doxycycline. It exhibited resistance to cefixime, sulfamethazine, and sulfafurazole.

### 3.8. Physiological and Biochemical Characteriation of the Bacterial

The physiological and biochemical characterization of isolate JXMa251025 is, shown in Table 4. Esculin, Urea, Glucose, Citrate, Lysine, Ornithine and Catalase were positive. Tests for Sorbitol, Mannitol, Arabinose, fructose, Galactose, Lactose, Sucrose, Maltose, Gelatin and Nitrate reduction were negative. According to the morphological and biochemical results, strain JXMa251025 was identified tentatively as *Nocardia seriolae*.

### 3.9. Phylogenetic Analysis

**Figure 7:**
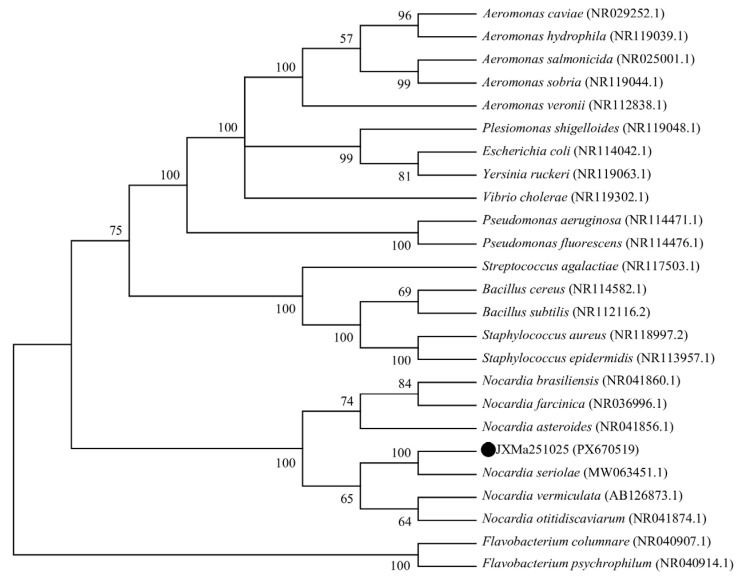
Phylogenetic tree of JXMa251025 isolation based on 16S rRNA gene sequences. The phylogenetic tree was constructed using the Neighbor-Joining method in MEGA V7.0, incorporating sequences from representative strains of related taxa. Bootstrap values (expressed as percentages) based on 2000 replicates are shown at the nodes. Black dots (●) indicate the JXMa251025 strain isolated in this study.

A 1436 bp sequence was amplified using the universal primers 27F and 1492R. A phylogenetic tree based on 16S rRNA sequences was constructed using MEGA V7.0 software. The results showed that the JXMa251025 strain (PX670519) and *Nocardia seriolae* (MW063451.1) clustered into the same clade (Figure 7).

**Figure 8:**
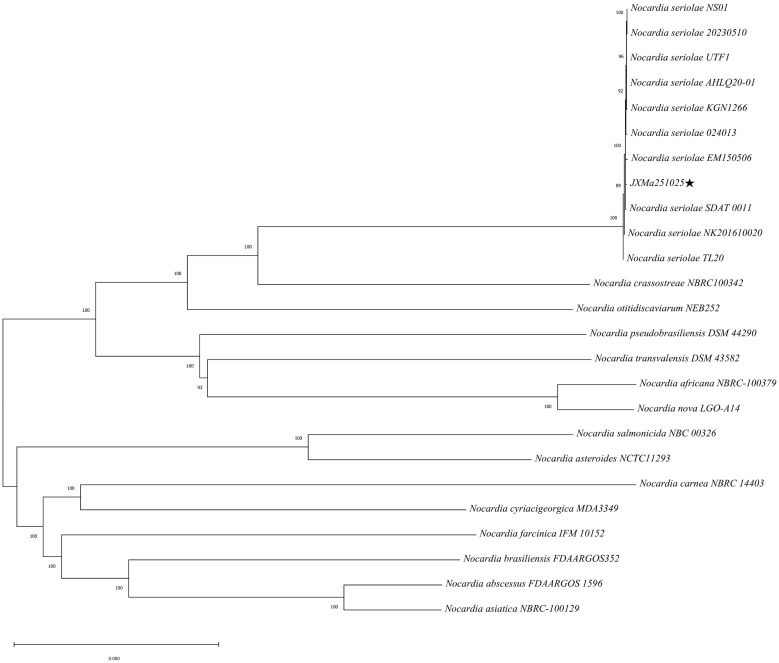
The GBDP phylogenetic tree constructed based on the whole-genome sequence. The black pentagram (★) indicates the strain JXMa251025 isolated in this study.

A GBDP phylogenetic tree of the whole genome of strain JXMa251025 was constructed via the TYGS platform (https://tygs.dsmz.de/) using FastME 2.1.6.1. The strain exhibited the highest similarity of 99.98% with *Nocardia gilthead seabream* SDTA-0011 (CP134713.1). This close phylogenetic relationship was further confirmed: strain JXMa251025 consistently clustered with known *Nocardia seriolae* (Figure 8).

**Figure 9:**
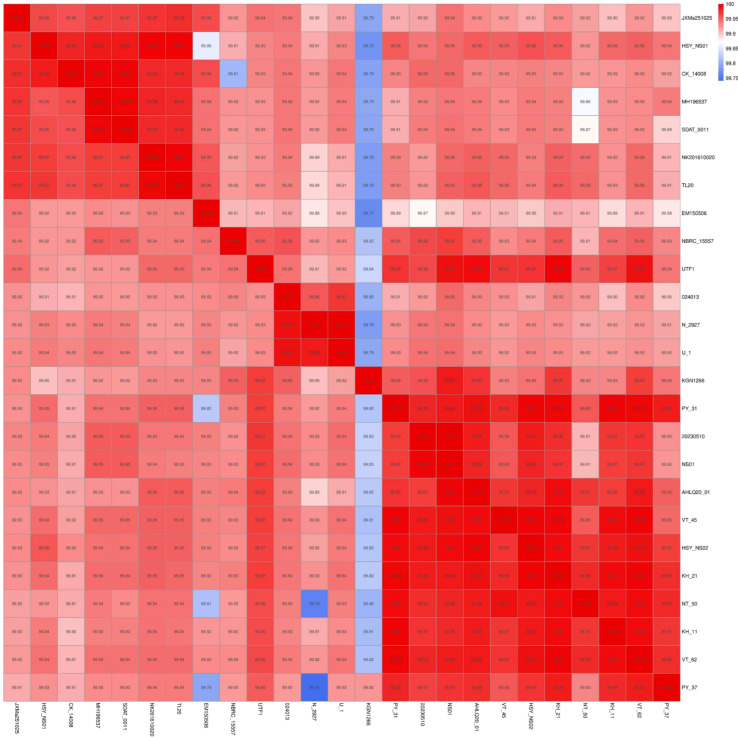
Phylogenetic analysis and gene distribution heatmap of 24 *Nocardia* species based on whole-genome sequences.

The whole-genome sequence of *Nocardia seriolae* strain JXMa251025 has been deposited in GenBank number CM150781.1. In addition, the taxonomic assignment of strain JXMa251025 as *Nocardia seriolae* was unequivocally confirmed by Average Nucleotide Identity (ANI) analysis. The results also showed that JXMa251025 exhibited ANI values ranging from 99.00% to 99.98% against 24 publicly available *Nocardia seriolae* genomes, with the highest identity (99.98%) observed against *Nocardia seriolae* SDTA-0011 (CP134713.1) (Figure 9). All ANI values significantly exceeded the established species delineation threshold of 95–96%, providing definitive genomic evidence for its classification within *Nocardia seriolae*.

### 3.10. Pathogenicity Analysis

**Figure 10:**
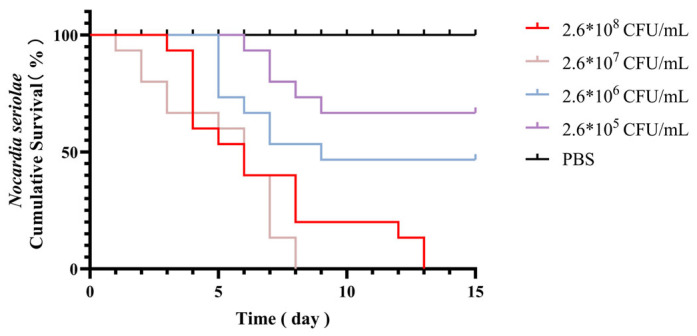
Concentration-dependent pathogenicity of the JXMa251025 strain in Chinese rice-field eels.

To confirm the pathogenicity of JXMa251025, an infection experiment was conducted using Chinese rice-field eels. During the 15-day post-infection period, fish in the control group exhibited no symptoms and showed a 100% survival rate. In contrast, infection groups inoculated with bacterial concentrations ranging from 2.6 × 10^5^ to 2.6 × 10^8^ CFU/mL displayed dose-dependent morbidity, mortality and variation in time to death. Specifically, all fish in the groups receiving 2.6 × 10^8^ CFU/mL and 2.6 × 10^7^ CFU/mL died by day 8 and day 13, respectively. Meanwhile, survival rates in the groups inoculated with 2.6 × 10^5^ CFU/mL and 2.6 × 10^6^ CFU/mL were 64% and 49%, respectively. Based on these data, the LD_50_ of JXMa251025 was calculated to be 9.76 × 10^5^ CFU/mL (Figure 10). PCR analysis confirmed the presence of JXMa251025 in challenged and dead fish, while no amplification was detected in mock-infected controls (Figure S1). These results unequivocally demonstrate that JXMa251025, isolated from diseased Chinese rice-field eels, is the causative agent of the disease observed in farmed populations.

## 4. Discussion

The genus *Nocardia*, within the family *Nocardiaceae*, encompasses aerobic, Gram-positive, filamentous, and partially acid-fast bacteria that are widely distributed as environmental saprophytes in soil and aquatic habitats [19,20]. Among the over 113 identified species, more than 30 are recognized as opportunistic pathogens, capable of causing nocardiosis in immunocompromised humans and a broad spectrum of animals, including companion animals, livestock, and aquatic species [21,22,23]. In aquaculture, nocardiosis represents a serious chronic systemic disease, with significant economic losses attributed to pathogens such as *Nocardia asteroides*, *Nocardia salmonicida*, and *Nocardia seriolae* [24,25,26]. Infections have been reported in various farmed fish, including Chinook salmon (*Oncorhynchus tshawytscha*), largemouth bass (*Micropterus salmoides*), and greater amberjack (*Seriola dumerili*), often resulting in substantial mortality [27,28,29]. Clinically, affected fish typically exhibit external lesions such as skin ulcers and gill tubercles, while internal pathology is characterized by granuloma formation in visceral organs, particularly the liver, kidney, and spleen [30,31]. This study reports, for the first time, a large-scale mortality event in farmed Chinese rice-field eels (*Monopterus albus*) caused by *Nocardia seriolae* strain JXMa251025, thereby extending the known host range of this pathogen beyond traditionally affected marine and freshwater fish [27,29]. Gross and histopathological examinations revealed typical signs of systemic nocardiosis, including multifocal visceral granulomas with central necrosis, hepatocyte degeneration, renal tubular necrosis, and splenic hemosiderin deposition—findings that align closely with previous descriptions in species such as sea bass (*Lateolabrax japonicus*) and hybrid snakehead [22,32]. The consistency in histopathological manifestations across different host species underscores a conserved host response, primarily involving a pyogranulomatous reaction aimed at containing the pathogen. This recurrent pathological pattern, also observed in other fish like groupers and amberjack [32,33], suggests that the inability to fully eliminate the bacterium within granulomas may contribute to chronic infection and systemic dissemination, as demonstrated in our experimental challenge with this strain. The emergence of *Nocardia* seriolae in Chinese rice-field eels, a freshwater teleost, represents a new host record and indicates the adaptive potential of this pathogen to diverse aquaculture environments. The method for bacterial identification involves a polyphasic approach that integrates morphological, physiological, and molecular characteristics, such as 16S rRNA and multilocus sequence analyses, to ensure taxonomic precision [33,34,35]. Recent advances in next-generation sequencing have further transformed microbial diagnostics by enabling comprehensive whole-genome analyses. In the current study, strain JXMa251025 was isolated from diseased Chinese rice-field eels in aquaculture facilities in Jiangxi. The identification of the strain as *Nocardia seriolae* was established through both molecular and genomic approaches. 16S rRNA gene sequencing demonstrated greater than 99% sequence homology with known *Nocardia seriolae* strains, while whole-genome sequencing further confirmed its classification by yielding an average nucleotide identity (ANI) of 99.98% when compared to *Nocardia seriolae* reference genomes, a value substantially surpassing the recognized 95–96% threshold for species delineation. The assembled genome displayed a typical *Nocardia* architecture, consisting of a single circular chromosome of 8.30 Mb with a GC content of 68.1%. These genomic characteristics are consistent with previously reported *Nocardia seriolae* genomes, which generally range from 7.70 to 8.37 Mb with a GC content around 68.1% [36,37,38]. Phylogenetically, JXMa251025 clustered with other *N. seriolae* strains. Its unique genomic islands and prophages may be related to adaptation to a freshwater host, and similar host-jump events have been reported in other aquatic pathogens [39].

The pathogenicity of *Nocardia* is closely associated with its virulence gene profile, and specific gene types directly determine the pathogenicity potential and infection severity [40,41,42]. In our study, the genome of strain JXMa251025 contains 253 putative virulence-associated genes. Among these, iron acquisition systems—including HSI-I, FbpABC, and several siderophore biosynthesis clusters—were identified. Similar siderophore systems have been characterized in other *Nocardia* species such as *N. farcinica* and *N. cyriacigeorgica* [43], and their presence in JXMa251025 is noted. The genome also encodes secretion system components (ESX-1, T3SS) and surface-associated factors (capsule, Antigen 85), which have been previously implicated in *Nocardia* pathogenesis [19,44]. Whether these systems are functionally active in JXMa251025 requires experimental validation in the future.

The high virulence of JXMa251025 was also experimentally confirmed, with an LD_50_ of 9.76 × 10^5^ CFU/mL in Chinese rice-field eels. This pathogenic potential is consistent with reports of *Nocardia seriolae* in other fish hosts [22,45]. For instance, the species has been associated with significant mortality in yellowtail (*Seriola quinqueradiata*) and sea bass (*Lateolabrax japonicus*), where cumulative mortality reached 60–70% in experimental challenges [22,45]. The acute mortality observed in our high-dose infection trials parallels findings in hybrid snakehead infected with a virulent *Nocardia seriolae* strain [32]. This phenotypic conservation suggests a common mechanism of infection and disease progression, likely driven by the core suite of virulence factors identified in the JXMa251025 genome.

Genomic analysis of strain JXMa251025 predicted a diverse set of antibiotic resistance genes (ARGs), including determinants associated with macrolides (e.g., erm, mef) and glycopeptides (e.g., van). However, phenotypic susceptibility testing revealed that the strain remains susceptible to all seven clinically tested antibiotics. This discrepancy demonstrates that the genomic presence of ARGs does not necessarily predict phenotypic resistance, a finding consistent with previous observations [46,47]. Importantly, the observed susceptibility to fluoroquinolones and amikacin aligns with earlier reports on antibiotic efficacy against aquatic nocardiosis [28,29]. Collectively, these results reinforce the importance of integrating conventional phenotypic susceptibility testing with genomic surveillance to inform therapeutic strategies and monitor resistance dynamics.

## 5. Conclusions

In conclusion, this study identifies a *Nocardia seriolae* strain JXMa251025 as the causative agent of mortality in farmed Chinese rice-field eels through pathological, microbiological, whole genomic sequence, and experimental infection experiments. Our findings demonstrate both common features of aquatic nocardiosis, including typical histopathology and conserved virulence-related genes, and also document a new host record, with this pathogen being identified in Chinese rice-field eels for the first time. The genomic characteristics, putative virulence-related genes and antimicrobial susceptibility profiles provide useful information for the diagnosis and control of this disease in Chinese rice-field eel aquaculture. This study showed the dynamic nature of aquatic animal diseases in the face of expanding and intensifying aquaculture, emphasizing the need for continuous surveillance, prompt diagnosis, and sustainable disease control practices.

## Figures and Tables

**Table 1:** Primers used in this study.

Primer Name	Sequences (From 5′ to 3′)	Reference
16s-27F	AGAGTTTGATCCTGGCTCAG	[17]
16s-1492R	TACGGCTACCTTGTTACGACTT	
NOC-*gntR*F	CCTGCACATGGCCTCGTTCT	[18]
NOC-*gntR*R	AGCGACTGGGTGAGTTCCTG	

**Table 2:** Collection and host information of other isolates of *Nocardia seriolae*.

Strains	Collection Date	Isolation Region	Host	GenBank
HSY−NS01	2014	China	*Channa Argus*	——
CK−14008	2014	South Korea	*Channa Argus*	——
MH196537	2018	South Korea	*Anguilla Japonica*	CP059737.1
SDAT−0011	2019	China	Fish	CP134713.1
NK201610020	2013	China	Hybrid Snakehead	CP063662.1
TL20	2021	China	Fish	CP073655.1
EM150506	2015	South Korea	*Anguilla Japonica*	CP017839.1
NBRC−15557	2019	Japan	Yellowtai	——
UTF1	2016	Japan	*Seriola Quinqueradiata*	AP017900.1
024013	2002	Japan	*Seriola Quinqueradiata*	AP028459.1
N−2927	2007	Japan	Yellowtai	——
U−1	2011	Japan	Yellowtai	——
KGN1266	2012	Japan	*Seriola Dumerili*	AP028458.1
PY_31	2014	Viet Nam	*Trachinotus Falcatus*	——
20230510	2023	China	*Siniperca Chuatsi*	CP130742.1
NS01	2022	China	*Micropterus Salmoides*	CP147977.1
AHLQ20−01	2020	China	*Micropterus Salmoides*	CP076862.1
VT_45	2015	Viet Nam	*Trachinotus Falcatus*	——
HSY−NS02	2018	China	*Micropterus Salmoides*	——
KH_21	2014	Viet Nam	*Trachinotus Falcatus*	——
NT_50	2014	Viet Nam	*Trachinotus Falcatus*	——
KH_11	2014	Viet Nam	*Trachinotus Falcatus*	——
VT_62	2015	Viet Nam	*Trachinotus Falcatus*	——
PY_37	2014	Viet Nam	*Trachinotus Falcatus*	——

**Table 3:** Detection of drug sensitivity of the JXMa251025 isolation.

Antibiotics	Content (μg·disc^−1^)	Inhibition Zone/mm	Sensitivity
Ciprofloxacin	5	30	S
Cefixime	5	0	R
Neomycin	30	20	S
Enrofloxacin	5	28	S
Florfenicol	30	30	S
Gentamicin	10	20	S
Amikacin	30	30	S
Sulfamethazine	300	0	R
Doxycycline	30	20	S
Sulfafurazole	2	0	R

Notes: “S” = Susceptible (diameter > 15 mm); “I” = Intermediate (10 mm < diameter ≤ 15 mm); “R” = Resistant (diameter ≤ 10 mm).

**Table 4:** Physiological and biochemical identification of JXMa251025 isolation.

Reaction Item	JXMa251025 Strain Result	*Nocardia seriolae* Result
Esculin	+	+
Sorbitol	−	−
Mannitol	−	−
Urea	+	+
Arabinose	−	−
fructose	−	−
Galactose	−	−
Lactose	−	−
Sucrose	−	−
Glucose	+	+
Maltose	−	−
Citrate	+	+
Lysine	+	+
Ornithine	+	+
Gelatin	−	−
Catalase	+	+
Nitrate reduction	−	−

Notes: “+” means positive reaction; “−” means negative reaction.

## Data Availability

The data presented in this study are available on request from the corresponding authors.

## Supplementary Materials

The following supporting information can be downloaded at: https://www.mdpi.com/article/10.3390/ani16081160/s1. Figure S1. Specific Detection of Nocardia seriolae in Tissues of Chinese rice-field eels Artificially Infected with Strain JXMa251025. A 249 bp specific fragment was amplified based on the gntR gene of Nocardia seriolae. M: DNA Marker; 1–3: Infected samples; 4: Positive control; 5: Negative control.
