# Supplementary figures and images for: Isolation, Identification and Whole-Genome Sequencing of a Nocardia seriolae Strain from Farmed Chinese Rice-Field Eels (Monopterus albus)
Source: Animals (Basel). 2026 Apr 10;16(8):1160. doi: 10.3390/ani16081160 (PMC13113822; doi:10.3390/ani16081160)

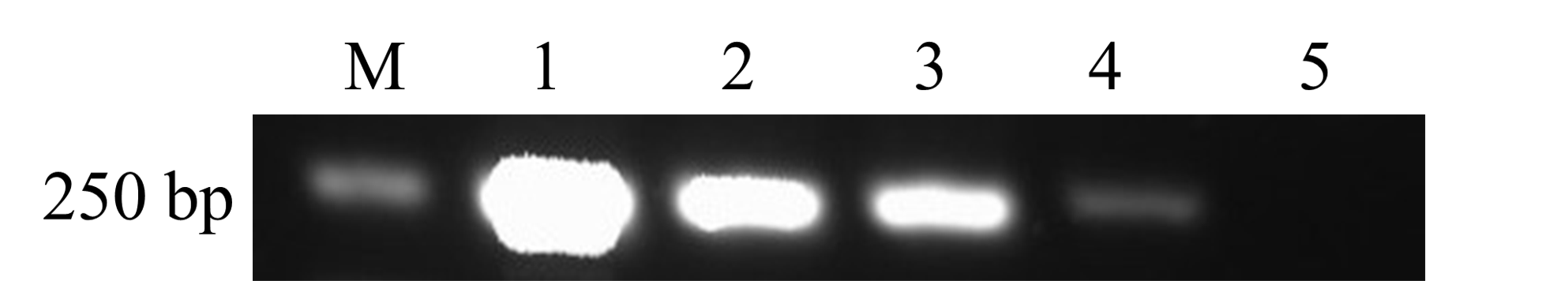

Supplement: Supplementary file 1 [file animals-16-01160-s001.zip › Figure S1.tiff]
